# P58 - The role of osteopontin and vitamin D in school-age asthmatic children for predicting asthma exacerbation

**DOI:** 10.1186/2045-7022-4-S1-P113

**Published:** 2014-02-28

**Authors:** Pınar Uysal, Meral Karaman, Özkan Karaman, Zeynep Tuba Arıkan Ayyıldız, Nevin Uzuner

**Affiliations:** 1 Adnan Menderes University Pediatric Immunology and Allergy, Aydın, Turkey; 2 Dokuz Eylul University Multidisiplinary Laboratory, Izmir, Turkey; 3 Dokuz Eylul University Pediatric Immunology and Allergy, Izmir, Turkey

## Background

Vitamin D plays an essential role in asthma by cell proliferation, differentiation and immunomodulation, and Osteopontin (OPN) is also associated with airway remodeling and fibrosis. Previously, it was shown that active Vitamin D and Vitamin D receptor cooperate in the transcriptional regulation of OPN expression.

## Objective

We aimed to evaluate the role of serum OPN and its association with 25-OH Vitamin D [25(OH)D] levels in asthmatic children and to investigate the possible role of associations during non-exacerbation and exacerbation periods and to compare the results with controls.

## Methods

In prospective, cross-sectional designed study, moderate-severe 85 asthmatic children and 60 healthy children were recruited. The severity of asthma was evaluated according to the GINA guideline. The 25(OH)D and OPN levels of asthmatics were investigated in non-exacerbation period, exacerbation period and compared with controls.

## Results

OPN levels (ng/ml) were significantly higher in asthmatics in exacerbation period when compared to non-exacerbation period, and controls [30,61±3,21 vs. 28,7±3,71 vs. 27,62±3,91, respectively; p<.001]. There was no significant difference among OPN levels between asthmatics and controls, (p=.094). 25(OH)D levels (ng/ml) were significantly lower in asthmatics in exacerbation period when compared to non-exacerbation period, and controls [30,15±5,11 vs. 32,23±5,80 vs. 34,16±5,86, respectively; p<.001]. There was no significant difference among 25(OH)D levels between asthmatics and controls, (p=.051).There was a correlation for both OPN and 25(OH)D levels among asthmatics in exacerbation period and non-exacerbation period, respectively (r=0,40, p<,001 and r=0,62, p<.001). But, no correlation between OPN and 25(OH)D levels among asthmatics in exacerbation and non-exacerbation periods (r=-0,78, p=,481; r=0,10, p=,448, respectively).

## Conclusion

OPN might play a role in childhood asthma and can be a useful marker for prediction of exacerbation. There was no correlation between OPN and 25(OH)D levels in asthmatic children. We suggest some other factors might have a role in OPN secretion besides vitamin D.

